# Continuous renal replacement therapy for lithium toxicity: A worthy treatment to avoid intradialytic hypotension and vasopressors

**DOI:** 10.1002/ccr3.8775

**Published:** 2024-04-23

**Authors:** Pooya Zardoost, Zachary Buckman, Joseph Weaver, Sidney Elston, Alex Prouty, Ryan Stuart, Henry L. Wehrum

**Affiliations:** ^1^ OhioHealth‐Doctors Hospital Columbus Ohio USA

**Keywords:** cardiovascular disorders, critical care medicine, general medicine, nephrology, psychiatry

## Abstract

While intermittent hemodialysis (HD) is the most efficient method of removing lithium in patients with lithium toxicity, continuous renal replacement therapy is an acceptable alternative in the setting of intradialytic hypotension. This form of dialysis can reduce the need for vasopressors during HD, which increases mortality.

## 1 INTRODUCTION

Lithium was first introduced in the 1970s for treatment of mania and continues to be a first‐line treatment even with the advent of new mood stabilizers, particularly for manic episodes in bipolar disorder as well as maintenance in patients with a history of manic episode.^1^, ^2^ Because it is readily diffused, intermittent hemodialysis (HD) is the preferred modality in the setting of toxicity.^1^, ^2^, ^3^ However, the degree of lithium toxicity itself along with hemodialysis can predispose patients to cardiovascular collapse.^3^ There is also a concern of rebound increases in lithium concentration due to lithium's high volume of distribution in muscle and nerve cells.^3^ In 2015, a systematic review conducted by the EXtracorporeal TReatments In Poisoning workgroup (EXTRIP) supported continuous renal replacement therapy (CRRT) as an acceptable alternative.^4^ However, many providers still hesitate to switch to this modality in the setting of intradialytic hypotension (IDH) given the slower rate of removal in CRRT. Intermittent HD in hemodynamically unstable patients leads to the use of vasopressors which is a risk factor for death in critically ill patients.^5^ We present a case where a patient presented with lithium intoxication and developed hypotension on intermittent HD. The critical care team was considering resuming HD with vasopressor therapy if needed, but with nephrology's input agreed to trial CRRT. She was successfully switched to this modality of dialysis, and her lithium levels and clinical symptoms resolved within days without any complications.

## 2 CASE REPORT

**FIGURE 1:**
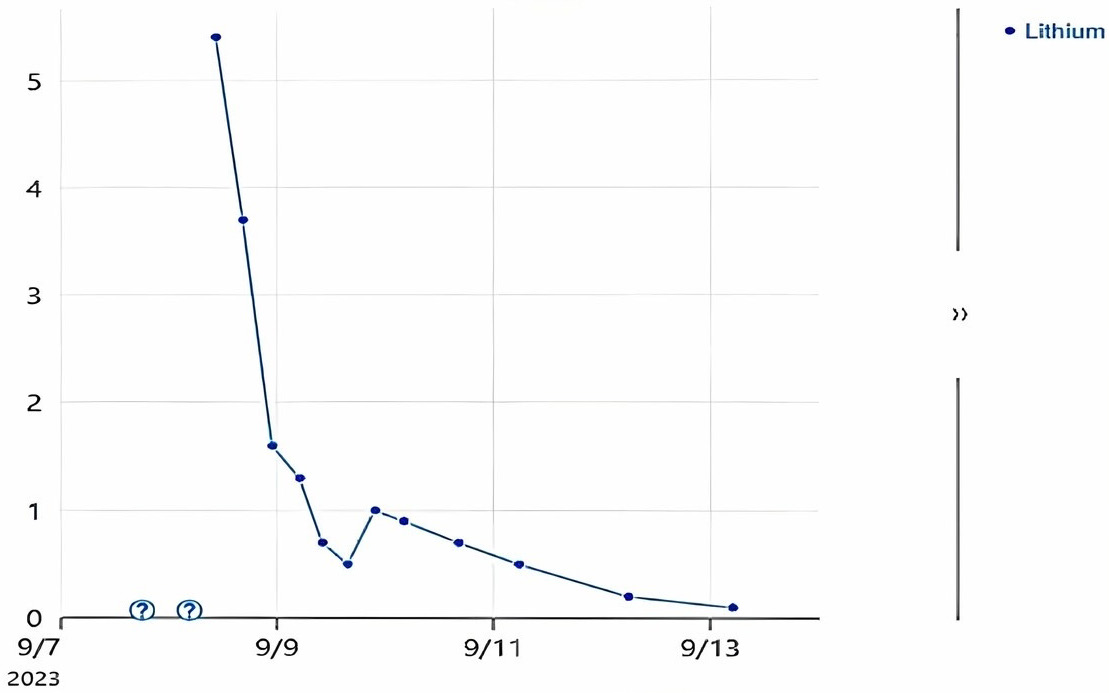
Patient's lithium concentration throughout admission. Initial lithium level in our facility was read as “greater than 6 mmol/L" in our electronic health record. HD was halted on 9/8 after 150 min, and the patient was transitioned to CRRT later than morning for a total of 28 h. Of relevance is a temporary jump in lithium levels after CRRT stopped, due to the rebound phenomenon.^6^

A 54‐year‐old woman with a history significant for bipolar 1 disorder and post‐traumatic stress disorder presented with suicidal ideation and intentional overdose, reporting that approximately 3 h before arrival to the hospital she took 180 tablets of 450 mg lithium, 5 tablets of 5 mg oxycodone, and 20 tablets of acyclovir of an unknown dose. Home medications included lamotrigine 100 mg twice daily, lithium 450 mg twice a day, quetiapine 400 mg at night, risperidone 2 mg daily, and sertraline 50 mg daily. She reported regret after this and immediately called medics. The patient's symptoms included abdominal pain and nausea with two episodes of vomiting. Vitals were 129/82 mmHg, 99 bpm, 15, 97.6°F, and 97% on room air. On the physical exam she was alert and oriented with no acute distress, but her speech was slurred with some intermittent confusion. Cardiovascular exam revealed a regular rate and rhythm, with brisk capillary refill. Electrocardiogram (EKG) revealed normal sinus rhythm and prolonged QTc at 520 ms. Laboratory values revealed a creatinine of 1.1 mg/dL and estimated glomerular filtration rate (eGFR) of 60 mL/min/1.73 m^2^, with an unknown baseline. The patient had several urinary occurrences throughout admission and denied changes in urine output. Abdominal exam was non‐tender with normal bowel sounds, and respirations were clear to auscultation and unlabored. Initial lithium level was reported as “greater than 6” mmol/L (Figure 1).

## 3 METHODS

The patient was started on 0.9% normal saline at 200 mL/h with plans for dialysis per nephrology's recommendations. A double lumen right femoral dialysis catheter was successfully placed, and the patient underwent intermittent hemodialysis. However, soon after her blood pressure dropped to 90/51 mmHg and HD was halted. She was placed on a short course of low‐dose norepinephrine infusion which was stopped after her blood pressure improved. The critical care team was initially considering resuming intermittent HD with reinitiation of vasopressors if necessary. After further discussions with nephrology, CRRT was chosen as the ultimate treatment. She was transitioned to CRRT at net even ultrafiltration rate, at 150 mL/min and dialysate flow of 1500 mL/h to enhance solute clearance while maintaining hemodynamic stability. The patient's blood pressure remained stable, and CRRT continued for a total of 28 h and was discontinued when lithium reached a level below 1.0 mmol/L. Subsequent EKG showed resolution of prolonged QTc. Her creatinine decreased to 0.66 mg/dL and eGFR increased to 104 mL/min/1.73 m^2^.

## 4 CONCLUSION AND RESULTS

The patient's symptoms resolved and with behavioral health's guidance she was transferred to a tertiary care center for inpatient psychiatric care. Her medications were adjusted with home lithium discontinued and replaced with divalproex 250 mg daily.

## 5 DISCUSSION

Lithium toxicity directly affects muscle and nerve cells, which include cardiomyocytes. Clinical manifestations include decreased level of consciousness, seizures, fatal arrhythmias, and impaired kidney function.^7^ Our patient's reading of “above 6 mEq/L” placed her well into the category of severe toxicity, which is seen at levels above 3.5 mEq/L.^7^ Furthermore, while our patient was only intermittently confused, her improvement in creatinine by more than 0.3 mg/dL after the clearance of her lithium levels indicated that the patient had acute kidney injury despite not knowing a prior baseline.^8^ Even more concerning was her prolonged QTc, which raised concern for the developing EKG signs of lithium toxicity that include bradycardia, flattened or inverted T waves, heart block, and sick sinus syndrome.^1^ The mechanism of action of lithium is unknown but is postulated to include modification of sodium transport in nerve and muscle cells and altering the metabolism of catecholamines and serotonin.^1^ High levels of lithium in the serum are either due to excess intake or reduced excretion.^1^, ^2^ Toxic levels are when the serum level is above 2 mEq/L, which is a narrow window above the acceptable chronic treatment range of 0.6–1.2 mEq/L.^1^ Causes of lithium toxicity include excessive intake due to suicidal intent or accidental overdose, or from impaired excretion by decreasing the urinary concentrating capacity of the kidney due to precipitation of nephrogenic diabetes insipidus.^2^ Physiological states that lead to sodium and water depletion such as vomiting, diarrhea, febrile illness, renal insufficiency, excessive exercise, or congestive heart failure can enhance lithium reabsorption in the kidneys.^2^

While the cause of our patient's lithium excess was intentional overdose, it is possible that other medications interactions played a role in the level of toxicity. Medications commonly reported to increase lithium levels include diuretics, nonsteroidal anti‐inflammatory drugs (NSAIDs), angiotensin‐converting enzyme inhibitors (ACEI), metronidazole, carbamazepine, phenytoin, and methyldopa.^1^ However, any medication that is renally cleared has also been reported to contribute. Of the medications our patient was on chronically as well as ingested on admission, acyclovir levels are renally excreted and others such as risperidone are increased in impaired renal function.^9^, ^10^, ^11^, ^12^, ^13^, ^14^ It is possible that excess acyclovir caused some renal impairment which in turn compounded the serum levels of lithium in our patient's serum.

While normal saline is often used to help enhance urinary excretion, her renal impairment combined with her lithium levels greater than 5 mEq/L were indications to consider extracorporeal treatment, of which hemodialysis is the most favored choice.^7^ Lithium has a small size of 74 Da, is rapidly absorbed and is not protein bound, making it easily diffusible through any modern filter.^6^, ^7^ Intermittent hemodialysis (HD) is preferred and is the most effective method of removal, influencing the multidisciplinary team's consideration of resuming this modality.^5^ Blood lithium levels tend to decline rapidly after the initiation of hemodialysis but can increase as re‐equilibration from the extracellular site takes place.^6^ This is known as the rebound phenomenon. To control this phenomenon, dialysis should be repeated until blood lithium levels remain below 1.0 mmol/L for 6–8 h.^5^ Continuous veno‐venous hemofiltration specifically has also been considered as a method for reducing the danger of rebounding serum lithium levels.^14^ While our patient did have some level of rebound with this method of dialysis as shown in Figure 1, she clinically improved, nevertheless.

While lithium toxicity has a risk of cardiovascular collapse, intermittent hemodialysis also has a risk of hypotension, especially due to its maximum clearance of 240 mL/min, as opposed to CCRT's maximal clearance of 80 mL/min.^7^ Our patient's level of lithium toxicity was high enough to precipitate hypotension with the added stress of intermittent hemodialysis. While intermittent HD is three times more efficient, CRRT has been studied in the last decade as an acceptable alternative in the setting of hemodynamic instability.^4^ To maximize clearance, it is recommended that the CRRT dose be above the standard 20–25 mL/kg dose that is usually prescribed for acute kidney injury. The patient was prescribed at a slightly higher dose of 27 mL/kg due to concerns of hemodynamic instability, but nevertheless had excellent clearance the day after, decreasing from a level of 3.7–1.3 mmol/L. The consideration and acceptance of this method of extracorporeal removal in the setting of IDH deserves more comfort among intensivists and nephrologists, especially if it can protect the patient from the adverse effects of vasopressors such as tissue necrosis, dysrhythmia, anxiety, and pulmonary edema.^15^

In the setting of severe lithium toxicity and intradialytic hypotension, CRRT should be considered among intensivists and nephrologists. Lithium toxicity itself has cardiovascular consequences and with the added stress of intermittent hemodialysis, this can cause a compounded effect on hemodynamic stability. The gentler clearance rate of CRRT has better hemodynamic tolerance and is an accepted alternative. Accepting this method of clearance among critical care providers and nephrologists can help protect the patient from adverse effects of vasopressors during intermittent HD, which increases mortality.

## AUTHOR CONTRIBUTIONS

**Pooya Zardoost:** Conceptualization; formal analysis; investigation; project administration; resources; supervision; visualization; writing – original draft. **Zachary Buckman:** Conceptualization; writing – review and editing. **Joseph Weaver:** Conceptualization; investigation; resources; writing – original draft. **Sidney Elston:** Resources; writing – original draft. **Alex Prouty:** Data curation. **Ryan Stuart:** Visualization. **Henry L. Wehrum:** Writing – review and editing.

## FUNDING INFORMATION

There is no funding to disclose for this case report.

## CONFLICT OF INTEREST STATEMENT

The authors have no conflicts of interest to disclose.

## CONSENT

Written informed consent was obtained from the patient to publish this report in accordance with the journal's patient consent policy.

## Data Availability

Data sharing is not applicable to this article as no new data were created or analyzed in this study.
